# Inflammatory Bowel Disease and Colorectal Cancer: Epidemiology, Etiology, Surveillance, and Management

**DOI:** 10.3390/cancers15164154

**Published:** 2023-08-17

**Authors:** Yoshihiro Sato, Shingo Tsujinaka, Tomoya Miura, Yoh Kitamura, Hideyuki Suzuki, Chikashi Shibata

**Affiliations:** Division of Gastroenterological Surgery, Department of Surgery, Tohoku Medical and Pharmaceutical University, Sendai 983-8536, Japan

**Keywords:** colorectal cancer, inflammatory bowel diseases, prognosis, surveillance

## Abstract

**Simple Summary:**

Although the incidence of inflammatory bowel disease (IBD)-related cancer has been decreasing, its prognosis remains worse than that of non-IBD-related cancers, owing to its multiple risk factors. This review explores the risk factors, epidemiology, surveillance strategies, and treatment recommendations for IBD-related cancers, as well as potential future research directions.

**Abstract:**

Patients with inflammatory bowel diseases (IBDs), such as ulcerative colitis and Crohn’s disease, have an increased risk of developing colorectal cancer (CRC). Although advancements in endoscopic imaging techniques, integrated surveillance programs, and improved medical therapies have contributed to a decreased incidence of CRC in patients with IBD, the rate of CRC remains higher in patients with IBD than in individuals without chronic colitis. Patients with IBD-related CRCs exhibit a poorer prognosis than those with sporadic CRCs, owing to their aggressive histological characteristics and lower curative resection rate. In this review, we present an updated overview of the epidemiology, etiology, risk factors, surveillance strategies, treatment recommendations, and prognosis of IBD-related CRCs.

## 1. Introduction

Patients with inflammatory bowel disease (IBD), including ulcerative colitis (UC) and Crohn’s disease (CD), have an increased risk of developing colorectal cancer (CRC) [1,2]. Although the incidence of CRC with IBD (IBD-CRC) is decreasing [3], its prognosis remains poorer than that of sporadic CRCs. Several distinct characteristics of IBD-CRC may contribute to this difference in prognosis, such as younger age at diagnosis, emergency presentation or diagnosis as an emergency, an increased likelihood of right-sided colon involvement [4], and histological features like multifocal tumors, poor/undifferentiated histology, or mucinous carcinomas [5,6]. Therefore, accurate risk stratification is crucial for effective personalized cancer surveillance by distinguishing between high-risk and low-risk individuals [7]. Advanced technologies during endoscopic surveillance programs provide valuable tools for diagnosing dysplastic and cancerous lesions [8,9,10], while new advanced techniques for endoscopic resection can also improve the prognosis of IBD-CRC [11].

In this comprehensive review, we present an updated overview of IBD-CRC, covering its epidemiology, etiology, and risk factors. We also emphasize the importance of individual risk stratification for recommending appropriate surveillance procedures and discuss both endoscopic and surgical management strategies for dysplasia. Furthermore, we discuss the short- and long-term oncologic outcomes of patients with IBD-CRC and potential future directions in the research field.

## 2. Epidemiology

The risk of CRC is significantly increased (by as much as two–three-fold) in patients with a long-standing history of UC and CD; however, the exact risk value may vary according to studies, time periods, and individual risk factors [3,12]. In a meta-analysis conducted by Eaden et al. in 2001, the overall prevalence of CRC in patients with UC was 3.2%, with cumulative risks of 2%, 8%, and 18% at 10, 20, and 30 years, respectively [13]. A subsequent study implied that the risk of CRC was low, with cumulative risks of 1%, 3%, and 7% at 10, 20, and 30 years, respectively [14]. Moreover, a decreased risk of CRCs was reported in 2014, with reported incidence rates of 0.91/1000, 4.07/1000, and 4.55/1000 patient-years (py) in the first, second, and third decades, respectively. This represents a decline from 4.29/1000 py in studies published in the 1950s to 1.21/1000 py in those published in the last decade [15]. As mentioned above, the incidence of IBD-CRC seems to decrease over time, likely due to improved medical therapies for inflammation management and endoscopic screening and surveillance [16,17]. From the perspective of the relative risk (RR) of developing CRCs in patients with IBD compared with that of the general population, the morbidity rates of those with IBD have remained higher. In a study conducted in 2020, the incidence rates of CRCs in the UC cohort and reference individuals were 1.29 and 0.82 per 1000 py, respectively. The mortality rate in the UC cohort was 0.55 per 1000 py compared with 0.38 per 1000 py in reference individuals during the same period [18].

## 3. Etiology and Molecular Mechanism

In patients with UC, the presence of mucosal inflammation is associated with an increased risk of subsequently developing colorectal neoplasia than of achieving mucosal healing [19]. In patients with chronic colitis, continuous stimulation of epithelial proliferation in an inflammatory process may lead to carcinogenesis [20,21]. It is well known that some specific molecular mediators promote IBD-CRC. Chronic inflammation causes oxidative stress-induced injuries [22]. Inflammation-driven carcinogenesis is characterized by a gradual increase in the levels of molecular markers associated with oxidative damage and DNA double-strand breaks [23]. Pathogenetic features such as chromosomal and microsatellite instabilities and DNA hypermethylation have been reported in patients with sporadic CRC as well as in those with IBD-CRC [20,24,25,26,27]. Both types of cancer share common functional driver genes, such as *APC*, *P53*, *MYC*, *KRAS*, *PIK3CA*, *SMAD4*, and *ARID1A* [28,29,30]. However, unlike sporadic CRC, IBD-CRC does not follow the conventional adenoma–carcinoma sequence; instead, it can progress from low- to high-grade dysplasia (HGD) and ultimately to CRC [31]. IBD-CRC has a different timing and frequency of genetic alterations as compared with sporadic CRC. In the carcinogenic process in IBD-CRC, mutation and loss of the *APC* gene are less frequent and occur in a later phase of the dysplasia–carcinoma sequence, whereas mutation and loss of *P53* are more frequent and likely to occur in an earlier phase [32]. Unlike sporadic CRC, these *P53* mutations can be observed in normal, non-dysplastic mucosa [2]. A recent meta-analysis showed that *P53* mutations were more common, but *KRAS* mutations were less frequent in patients with IBD-CRC. Moreover, both *KRAS* and *P53* mutations occur more frequently in patients with IBD-CRC than in those without IBD-related dysplasia. Accordingly, *P53* may play a more important role in the development of IBD-CRC [33]. Therefore, *KRAS* and *P53* mutations can be informative biomarkers of IBD-CRC, given the higher prevalence of these genetic alterations than that in non-dysplastic mucosa [34]. In addition to the known molecular risk factors above, risk stratification and prediction of cancer progression can be improved by the novel molecular biomarkers using gene panels, transcriptomics, and clonal evolutionary dynamics [35].

## 4. Risk Factors for Carcinogenesis

To minimize the consequences of IBD-CRC, it is crucial to accurately assess individual patient risks and identify relevant risk factors that necessitate frequent surveillance or intensive treatment. These risk factors can be classified into patient- and disease-related factors, with the duration and extent of chronic inflammation being reported as the most consistently associated factors [35,36,37,38].

### 4.1. Patient-Related Factors

UC diagnosis at a young age has been consistently associated with a greater risk of CRC than UC diagnosis at an older age [1], and longer disease duration has been reported to significantly increase the cumulative incidence of CRC [13,38,39]. Having a family history of IBD-CRC also significantly affects the incidence, with a two-fold higher risk of CRC in patients with first-degree CRC relatives than in those without first-degree CRC relatives [40]. A recent meta-analysis of 15 studies demonstrated that patients with a family history of CRC had an odds ratio (OR) of 2.62 (95% confidence interval [CI], 1.93–3.57) when compared with those without a family history [7]. Sex differences have also been proposed, with male patients exhibiting a higher risk of CRC than female patients, based on a pooled analysis of 60 studies (OR, 1.27; 95% CI, 1.12–1.44) [7]. This sex difference resembles that noted in sporadic CRC, and estrogen may have a preventive role in the development of CRC [41]. A concurrent diagnosis of primary sclerosing cholangitis (PSC) is one of the well-known risk factors for IBD-CRC [42]. Wijnands et al. reported a univariate OR of 4.14 (95% CI, 2.85–6.01) and a multivariate OR of 3.53 (95% CI, 1.83–6.79), and almost all independent analyses per study type indicated an elevated risk in patients with IBD and PSC [7]. For patients with both IBD and PSC, alterations in bile acid metabolism, intestinal/biliary microbiomes, and the systematic immune system are suggested as contributing factors for cancer development [36].

### 4.2. Disease-Related Factors

The wide extent of disease involvement is a strong risk factor for the development of IBD-CRC. In general, a wide extent of disease involvement is defined when >50% of the colonic area is affected in patients with CD or when mucosal inflammation extends proximally to the splenic flexure in patients with UC at any point during the disease course. A recent meta-analysis comprising 40 studies showed that the pooled univariate OR in patients with extensive UC was 2.43 (95% CI, 2.01–2.93) compared to the those with left-sided UC, while the pooled hazard ratio (HR) including three studies on UC cases was 3.48 (95% CI, 1.58–7.65) [7]. Patients with IBD who are previously diagnosed with low-grade dysplasia carry a high risk of CRC. The pooled univariate OR was 10.85 (95% CI, 5.13–22.97), from eight studies. Moreover, patients with IBD have post-inflammatory polyps, colonic stenosis, and severe inflammation on histology, all of which contribute to a higher risk in IBD patients with than in those without IBD [7]. The accumulation of these factors contributes to the inflammatory burden, which plays a critical role in the development of IBD-CRC [43].

## 5. Surveillance Strategy and Management

### 5.1. Surveillance Strategies

Colonoscopy is considered the fundamental tool for CRC surveillance in patients with IBD. The primary objective of this surveillance is to identify endoscopically removable premalignant lesions or early-stage CRC, leading to improved prognosis and treatment outcomes [44]. A recent meta-analysis, conducted in 2018, highlighted the importance of appropriate surveillance [45]. Bye et al. assessed the effectiveness of endoscopic surveillance in decreasing IBD-CRC-related mortality and found that the cancer detection rate was significantly higher in the non-surveillance group (3.2%) than in the surveillance group (1.8%) (OR, 0.58; 95% CI, 0.42–0.80; *p* < 0.001). Moreover, CRC-associated death was significantly lower in the surveillance group (8.5%, 15/176) than in the non-surveillance group (22.3%, 79/354) (OR, 0.36; 95% CI, 0.19–0.69; *p* = 0.002). In addition, the early-stage CRC detection rate was significantly higher in the surveillance group (15.5%) than in the non-surveillance group (7.7%) (OR, 5.4; 95% CI, 1.51–19.3; *p* = 0.009) [45]. Several international guidelines recommend active surveillance of patients with IBD to detect and resect dysplastic lesions before they progress to HGD or CRC [8,9,38,46,47,48]. Most guidelines advocate that all patients with colonic IBD undergo initial colonoscopy screening for dysplasia 8–10 years after the diagnosis of the disease. Continued surveillance colonoscopy is advised even if the disease is well-controlled, as chronic inflammation can lead to a false-positive pathological diagnosis of dysplasia [49]. Table 1 shows the recommended surveillance intervals after the initial colonoscopy, which are based on individual risk of CRC [38,44].

### 5.2. Recommended Endoscopic Techniques

To maximize the efficacy of endoscopic surveillance, optimized mucosal visualization and improved operator performance are crucial [50]. Recently, several new endoscopic techniques have emerged to identify dysplastic or cancerous lesions [8,9]. According to the SCENIC Consensus, high-definition (HD) white-light endoscopy (WLE) is preferred over standard-definition (SD) WLE for surveillance [48]. Several international societies provide guidance on techniques for surveillance colonoscopy (Table 2) [8,38,47,51,52]. A retrospective analysis compared 160 colonoscopies with SD-WLE and 209 colonoscopies with HD-WLE and showed that the colonoscopies with HD-WLE improved the targeted detection of dysplastic lesions during periodic surveillance [53]. Although HD-WLE can allow visualization of most forms of dysplasia, chromoendoscopy (CE) may further facilitate the detection of dysplasia [54]. Dye spray CE (DCE) using methylene blue or indigo carmine can help identify the areas of interest and distinguish the borders between the normal mucosa and the suspected lesions. Technical advancements in endoscopic imaging have developed virtual CE (VCE) that can be utilized without spraying dye agents. The American College of Gastroenterology (ACG) clinical guidelines advocate endoscopic surveillance with HD-WLE using narrow-band imaging (NBI) or DCE to identify dysplasia in patients with UC [8]. To date, several studies have compared endoscopic techniques; Clarke et al. performed a case-control study of adult patients with IBD to compare HD-WLC and DCE performance for the detection of dysplasia, showing a significantly increased rate of polyp detection in DCE over HD-WLC (1.35 vs. 0.80, *p* = 0.018) but no significant difference in the occurrence of dysplasia (10.2% vs. 6.7%, *p* = 0.39) or adenoma (10.2% vs. 9.0%, *p* = 0.31) [55]. A meta-analysis incorporating six randomized controlled trials (RCTs) and five prospective trials revealed that CE was better than WLE in diagnosing a larger number of patients with dysplasia (RR, 2.05; 95% CI, 1.62–2.61) and dysplastic lesions per patient (RR, 2.04; 95% CI, 1.40–2.98) [56]. This difference might be attributed to the disparate inclusion criteria and population diversity. El-Dallal et al. conducted a systematic review to evaluate the efficacy of VCE vs. HD-WLE or DCE, including 11 RCTs with 1328 patients. The efficacy of VCE did not statistically differ from DCE (risk ratio [RR], 0.77; 95% CI, 0.55–1.08) or HD-WLE (RR, 0.72; 95% CI, 0.45–1.15) in per-patient analysis. By contrast, VCE showed similar results compared with DCE (RR, 0.72; 95% CI, 0.47–1.11) but poorer results compared with HD-WLE (RR, 0.62; 95% CI, 0.44–0.88) in per dysplasia analysis [57]. Bisschops et al. performed a multicenter RCT to compare the diagnostic value of CE (using methylene blue) and VCE (using NBI) in 131 patients with intractable UC. No significant differences were observed in terms of the mean number of neoplastic lesions per colonoscopy (0.47 vs. 0.32, *p* = 0.992) or neoplasia detection rate (21.2% vs. 21.5%, *p* = 0.964) [58]. Given these published data, the American Gastroenterological Association (AGA) Expert Review recommends VCE and DCE for dysplasia detection in patients with IBD [8].

### 5.3. Management of Dysplasia

During surveillance colonoscopy, precancerous lesions have been identified as adenomatous polyps, dysplasia-associated lesions or masses, and flat dysplasia [59]. In 2015, the SCENIC international consensus described the term “lesions” based on the standard Paris classification [48]. The AGA Expert Review classified the lesions into polypoid (≥2.5 mm tall), non-polypoid (<2.5 mm), or invisible (detected on non-targeted biopsy) [38]. In patients with well-controlled inflammation, endoscopic resection of dysplastic lesions should be considered. Patients must provide informed consent, understanding the risks, complications, and possibility of surgery if incomplete endoscopic resection occurs [37]. Endoscopic mucosal resection and endoscopic submucosal dissection (ESD) are used for endoscopic resection. ESD allows *en bloc* resection of larger lesions with distinct margins. Nevertheless, ESD in patients with IBD can be challenging due to the presence of fibrosis and chronic inflammation [60]. Manta et al. analyzed 53 patients with UC who underwent ESD for visible dysplastic lesions, showing *en bloc* and R0 resection rates of 100% and 96.2%, respectively, with the detection of two metachronous lesions. Another systematic review, including six other studies, revealed that the *en bloc* resection rate was 88.4% for lesions and 91.8% for 208 patients, while the R0 resection rate was 78.2% for lesions and 81.3% for patients. Thus, this study suggested that ESD is a feasible treatment option for resecting non-invasive dysplastic lesions in patients with UC [61]. However, when invisible dysplasia is detected through random biopsies, the appropriate strategies to discuss include intense surveillance, reassessment by IBD experts, or surgical resection based on patient factors (such as age, family history, and concomitant PSC) and disease factors (such as severity of dysplasia, inflammation in the background mucosa, and uni- or multi-focality of the dysplasia) [37,38,44].

### 5.4. Chemoprevention of IBD-CRC

Various therapeutic agents have been employed in clinical practice to prevent carcinogenesis in patients with IBD [36,62]. Qiu et al. reported in their systematic review with meta-analysis that 5-aminosalicylic acid (5-ASA) demonstrated a chemopreventive effect on dysplasia/CRC in clinical-based studies. However, the effect was limited to patients with UC, not in those with CD [63]. According to the recent consensus guidelines, the BSG, the European Crohn’s and Colitis Organization (ECCO), and the Japanese Society of Gastroenterology (JSGE) have recommended the use of 5-ASA for chemoprevention [47,64,65]. The BSG also suggested the use of thiopurines with a weak recommendation [47], but the benefit must be weighed against the potential risk of thiopurines for secondary development of lymphoproliferative malignancies [62]. In contrast, protective roles of statins and ursodeoxycholic against CRC have been controversial. Folic acids might have a protective effect, although sufficient evidence has been still lacking. Biologic agents such as tumor necrosis factor-α (TNF-α), nonsteroidal anti-inflammatory drugs (NSAIDs), or acetylsalicyclic acid have failed to demonstrate protective effects [62]. Overall, mesalamine has solely been accepted as an effective chemopreventive agent, supported with strong recommendations by the current guidelines worldwide.

### 5.5. Surgical Management of IBD-CRC

Surgery is indicated for endoscopically unresectable dysplasia due to submucosal invasion, invisible dysplasia detected in random biopsy, or “high-risk” colons, such as those with HGD with PSC [48,66]. Total proctocolectomy with ileal pouch-anal anastomosis (IPAA) is the standard procedure for cases of HGD or CRC [59,67]. Stapled anastomosis is technically simple and superior to hand-sewn in terms of fewer anastomotic complications, better bowel function, and improved quality of life [68]. However, stapled anastomosis may carry the risk of neoplasia arising from a “cuff” of residual rectal mucosa. A systematic review that focused on pouch-related cancer showed that rectal mucosectomy did not eliminate subsequent dysplasia or cancer, but the incidence of cancer increased by eight times (OR, 8; 95% CI, 1.3–48.7) when mucosectomy was not indicated [69]. While total proctocolectomy is generally recommended, subtotal or partial colectomy are optional for patients with significant comorbidities, endoscopically unresectable unifocal neoplasia without other high-risk histological factors, and colonic CD without rectal involvement [37]. Dysplasia or cancer may arise from a diverted rectum or rectal stump left in situ [70]. Derikx et al. evaluated the risk of cancer after colectomy in their systematic review and meta-analysis, including 13 studies involving rectal stump surgery, 35 studies involving ileorectal anastomosis (IRA), and 33 studies of IPAA. The incidence of cancer was significantly increased in the rectal stump (2.1%) and IRA groups (2.4%) compared with that in the IPAA group (0.5%), and the OR was 6.4 (95% CI, 4.3–9.5, *p* < 0.001) [71]. Another recent systematic review involving 23 studies reported a pooled incidence of residual rectal carcinoma of 1.3%, with an incidence of 0.7% in patients with rectal stump surgery and 3.2% in those with IRA [72]. Bogach et al. evaluated the relationship between the surgical extent and prognosis in their population-based study. The 5-year survival rates were 63.7% in patients with UC and 57.5% in those with CD, and the multivariate analysis revealed that the survival outcome was inferior in patients who underwent total colectomy than in those who underwent segmental resection (HR, 1.70; 95% CI, 1.31–2.21; *p* < 0.001). No significant difference was observed between patients who underwent segmental resection and those who underwent proctocolectomy (HR, 0.99; 95% CI, 0.78–1.27) [73]. Adequate assessment of patients’ risk factors and reasonable decisions regarding surgical procedures are important for improving surgical outcomes in patients with IBD. Ramsay et al. reported a short-term outcome after CRC surgery between patients with IBD and those without IBD. Patients with IBD had increased postoperative complications (adjusted OR [AOR], 1.26; 95% CI, 1.06–1.50) including postoperative infection and deep vein thrombosis. Moreover, patients with IBD had a longer hospital stay (adjusted coefficient, 0.86 days; 95% CI, 0.42–1.30), received more blood transfusions (AOR, 1.59; 95% CI, 1.30–1.94), and experienced more readmissions within 30 days (AOR, 1.44; 95% CI, 1.01–2.04) than those without IBD. Therefore, the authors concluded that IBD could adversely affect outcomes after CRC surgery [74].

## 6. IBD-CRC Prognosis

IBD-CRC accounts for up to 15% of annual deaths in patients with IBD [75]. Generally, the prognosis of IBD-CRC is worse than that of sporadic CRCs. Mohan et al. performed a meta-analysis, including 18 studies with 1037 patients, and reported that the pooled risk (rate per 1000 py of follow-up) of CRC, HGD, and any lesion was 2 (95% CI, 0–3), 2 (95% CI, 1–3), and 43 (95% CI, 30–57), respectively [76]. However, conflicting results on long-term outcomes have been reported in recent studies. Lin et al. performed a population-based study and examined the long-term survival outcome of 222 patients with IBD-CRC (limited to UC) and 1110 patients with sporadic CRC. The disease-free survival rate was comparable between the IBD-CRC and sporadic CRC groups, with an HR of 1.06 (95% CI, 0.85–1.32), recurrence-free survival HR of 1.14 (95% CI, 0.86–1.53), and an overall mortality HR of 1.15 (95% CI, 0.89–1.48) [77]. Another population-based analysis conducted by Birch et al. showed that the patients with IBD-CRC were younger at cancer diagnosis (median; 66 vs. 72 years, *p* < 0.01), presented a tendency toward emergency cancer diagnosis (25.1% vs. 16.7%, *p* < 0.01), and had an increased prevalence of right-sided tumors (37.4% vs. 31.5%, *p* < 0.01) [4]. A recently conducted population-based study also demonstrated that patients with IBD-CRC had a significantly increased 2-year cancer-specific mortality rate compared to that of patients with sporadic CRC (HR, 1.35; 95% CI, 1.18–1.55) [78]. Lu et al. conducted a systematic review and meta-analysis and reported that patients with IBD-CRC had significantly decreased overall survival (HR, 1.33; 95% CI, 1.20–1.47) and cancer-specific survival (HR, 2.17; 95% CI, 1.68–2.78) compared with those with sporadic CRC. In addition, IBD-CRC exhibited better diagnosis of poorly differentiated adenocarcinomas (OR, 2.02; 95% CI, 1.57–2.61) and mucinous/signet ring cell carcinomas (OR 2.43; 95% CI, 1.34–4.42), synchronous (OR, 3.18; 95% CI, 2.26–4.47) and right-sided tumors (OR, 1.62; 95% CI, 1.05–2.05), male dominance (OR, 1.10; 95% CI, 1.05–1.16), and decreased R0 resection rate (OR, 0.60; 95% CI, 0.44–0.82). These data suggest that the worse prognosis of IBD-CRC may reflect the aggressive histological features and decreased resectability of the neoplastic lesions [79].

## 7. Conclusions and Future Directions

Over the past decade, significant advancements have been made, contributing to our understanding of the risk factors for carcinogenesis and the management of colorectal dysplasia in IBD. Identifying risk factors and high-risk patients is crucial for improving the prognosis of IBD-CRC. By enrolling high-risk patients into stratified surveillance programs, using advanced visualization techniques for surveillance colonoscopy, and determining the adequate timing of resection for detected dysplasia, the survival outcomes of patients with IBD-CRC can be improved. Furthermore, ongoing studies are necessary to improve our understanding of the mechanisms underlying IBD-CRC and identify novel therapeutic targets and interventions that can reduce the risk of carcinogenesis. The development of new biomarkers and diagnostic techniques can also aid in the early detection and monitoring of dysplasia. Through continuous studies in this field, we may further improve the outcomes of patients with IBD and ultimately mitigate the impact of IBD-CRCs on public health.

## Figures and Tables

**Table 1:** Recommended surveillance strategies based on risk stratification in patients with IBD.

Patients	High Risk	Intermediate Risk	Low Risk
Risk factors	Moderate or severe inflammation PSC Family history of CRC in FDRs aged < 50 years Dense pseudopolyposis <5-year history of invisible dysplasia or high-risk visible dysplasia	Mild inflammation Family history of CRC but no FDRs aged <50 years Previous episode of severe colitis <5-year history of invisible dysplasia or high-risk visible dysplasia <5-year history of low-risk visible dysplasia	Maintaining disease remission with mucosal healing plus either of ≥2 consecutive examinations without dysplasia Minimal colitis (ulcerative proctitis or <1/3 of the colon in CD)
Surveillance interval	1 year	2 or 3 years	5 years

CD, Crohn’s disease; CRC, colorectal cancer; FDR, first-degree relative; PSC, primary sclerosing cholangitis.

**Table 2:** Recommended techniques for surveillance colonoscopy.

Guidelines	Recommended Technique
ACG 2019 [8]	SD colonoscopy with DCE HD-WLE with NBI or DCE
AGA 2021 (clinical practice update) Expert consensus [37]	HD endoscopy SD-WLE with DCE HD-WLE with VCE, as an alternative to DCE
ESGE guideline 2019 [49]	HD endoscopy and DCE or VCE
BSG 2019 [46]	HD-WLE rather than SD-WLE SD or HD-WLE with DCE
AOCC and APAG, 2020 Expert consensus [50]	CE is preferred over WLE

ACG, American College of Gastroenterology; AGA, American Gastroenterological Association; AOCC, Asian Organization for Crohn’s and Colitis; APAG, Asia Pacific Association of Gastroenterology; BSG, British Society of Gastroenterology; CE, chromoendoscopy; DCE, dye spray chromoendoscopy; ESGE, European Society of Gastrointestinal Endoscopy; HD, high definition; SD, standard definition; VCE, virtual chromoendoscopy; WLE, white-light endoscopy.

## Data Availability

The data presented in this study are available in this article.
